# Comparative Transcriptome Analysis of Early- and Late-Bolting Traits in Chinese Cabbage (*Brassica rapa*)

**DOI:** 10.3389/fgene.2021.590830

**Published:** 2021-03-04

**Authors:** Xiaochun Wei, Md. Abdur Rahim, Yanyan Zhao, Shuangjuan Yang, Zhiyong Wang, Henan Su, Lin Li, Liujing Niu, Md. Harun-Ur-Rashid, Yuxiang Yuan, Xiaowei Zhang

**Affiliations:** ^1^Institute of Horticulture, Henan Academy of Agricultural Sciences, Zhengzhou, China; ^2^Department of Genetics and Plant Breeding, Sher-e-Bangla Agricultural University, Dhaka, Bangladesh

**Keywords:** comparative transcriptome, early bolting, late bolting, differentially expressed genes, Chinese cabbage

## Abstract

Chinese cabbage is one of the most important and widely consumed vegetables in China. The developmental transition from the vegetative to reproductive phase is a crucial process in the life cycle of flowering plants. In spring-sown Chinese cabbage, late bolting is desirable over early bolting. In this study, we analyzed double haploid (DH) lines of late bolting (“Y410-1” and “SY2004”) heading Chinese cabbage (*Brassica rapa* var. *pekinensis*) and early-bolting Chinese cabbage (“CX14-1”) (*B. rapa* ssp. *chinensis* var. *parachinensis*) by comparative transcriptome profiling using the Illumina RNA-seq platform. We assembled 721.49 million clean high-quality paired-end reads into 47,363 transcripts and 47,363 genes, including 3,144 novel unigenes. There were 12,932, 4,732, and 4,732 differentially expressed genes (DEGs) in pairwise comparisons of Y410-1 vs. CX14-1, SY2004 vs. CX14-1, and Y410-1 vs. SY2004, respectively. The RNA-seq results were confirmed by reverse transcription quantitative real-time PCR (RT-qPCR). A Kyoto Encyclopedia of Genes and Genomes (KEGG) pathway analysis of DEGs revealed significant enrichment for plant hormone and signal transduction as well as starch and sucrose metabolism pathways. Among DEGs related to plant hormone and signal transduction, six unigenes encoding the indole-3-acetic acid-induced protein ARG7 (BraA02g009130), auxin-responsive protein SAUR41 (BraA09g058230), serine/threonine-protein kinase BSK11 (BraA07g032960), auxin-induced protein 15A (BraA10g019860), and abscisic acid receptor PYR1 (BraA08g012630 and BraA01g009450), were upregulated in both late bolting Chinese cabbage lines (Y410-1 and SY2004) and were identified as putative candidates for the trait. These results improve our understanding of the molecular mechanisms underlying flowering in Chinese cabbage and provide a foundation for studies of this key trait in related species.

## Introduction

*Brassica* belongs to the Brassicaceae family, which consists of several economically important vegetable crops consumed worldwide (Cheng et al., 2014). Vegetables in this genus exhibits various morphotypes, including leafy heads (cabbage, *Brassica oleracea* var. *capitata*; Chinese cabbage, *Brassica rapa* var. *pekinensis*), enlarged inflorescences (cauliflower, *B. oleracea* var. *botrytis*; broccoli, *B. oleracea* var. *italica*), enlarged axillary buds (Brussels sprouts, *B. oleracea* var. *gemmifera*), enlarged stems (kohlrabi, *B. oleracea* var. *gongylodes*), and enlarged roots (turnip, *B. rapa* subsp. *rapa*) (Zhao et al., 2005; Cheng et al., 2014, 2016). Furthermore, these vegetables contain various health-promoting secondary metabolites, including glucosinolates, phenolics, carotenoids, flavonoids, and anthocyanins, which have protective effects against inflammation, cardiovascular diseases, and age-related diseases (Manchali et al., 2012).

*Brassica rapa* subspecies show high morphological diversity (Zhao et al., 2005). In particular, the heading Chinese cabbage (*B. rapa* L. subsp. *pekinensis*) is one of the most important *Brassica* vegetables in Asian countries, especially in China, Korea, and Japan (Bong et al., 2012; Cheng et al., 2014). It is a major vegetable crop produced in China, where it is considered as a key source of mineral nutrition (Wu et al., 2008). Moreover, Chinese cabbage is the main ingredient of the most popular traditional Korean side dish kimchi (Bong et al., 2012; Lee et al., 2014). The flowering Chinese cabbage (*B. rapa* ssp. *chinensis* var. *parachinensis*) produces elongated, tender, and thick stalks with rapid bolting as edible organs (Cheng et al., 2014; Huang et al., 2017). Pak choi (*B. rapa* subsp. *chinensis*) produces smooth dark green leaves with a prominent white midrib instead of forming a leafy head (Zhao et al., 2005).

In angiosperms, flowering is the most important developmental transition in the plant life cycle (Song et al., 2015). This transition is controlled by endogenous and environmental signals (Song et al., 2015; Zhang et al., 2015). More than 180 genes identified by functional analyses are associated with flowering time in *Arabidopsis* (Fornara et al., 2010). Several of these genes form a complex regulatory network involving six key pathways, including photoperiod, vernalization, ambient temperature, age, autonomy, and gibberellin pathways (Fornara et al., 2010; Zhang et al., 2015; Huang et al., 2017). However, photoperiod and vernalization related to day length and low temperatures, respectively, have also been identified as the major pathways for the regulation of flowering time (reviewed in Song et al., 2013). Several homologs of *Arabidopsis* genes related to flowering in *B. rapa* and other plant species have been identified (Andersen et al., 2004; Greenup et al., 2009; Cheng et al., 2011; Duan et al., 2015; Xu et al., 2015). Mutations in the *CONSTANS* (*CO*), *GIGANTEA* (*GI*), and *FLOWERING LOCUS T* (*FT*), related to the photoperiod pathway, result in delayed flowering, but short days do not affect flowering time, unlike wild-type *Arabidopsis* (Suárez-López et al., 2001; Simpson and Dean, 2002; Moon et al., 2003). The *CO* gene encodes a zinc finger transcription factor that triggers the transcriptional upregulation of downstream floral integrator genes, including *FT* and *SUPPRESSOR OF OVEREXPRESSION OF CO1* (*SOC1*), in leaves under long-day conditions (Samach et al., 2000; Srikanth and Schmid, 2011; Zhang et al., 2015). Furthermore, *FT* and *SOC1* act as activators of floral meristem identity genes, such as *LEAFY* (*LFY*) and *APETALA 1* (*AP1*) (Amasino, 2005). In *Arabidopsis*, *FRIGIDA* (*FRI*; encoding two coiled−coil motif-containing protein) and *FLOWERING LOCUS C* (*FLC*; encoding a MADS−box transcription factor) are involved in the annual winter habit (late flowering) (Michaels and Amasino, 1999; Johanson et al., 2000; Moon et al., 2003; Amasino, 2005). Mutations in these genes result in early flowering in *Arabidopsis* (Michaels and Amasino, 1999; Johanson et al., 2000; Choi et al., 2011). *FRI* positively regulates the transcription of the flowering repressor, *FLC* (Choi et al., 2011). In contrast, vernalization results in early flowering via suppressing the *FLC* (Michaels and Amasino, 1999; Sheldon et al., 1999). Three genes, *VIN3* (*VERNALIZATION INSENSITIVE 3*), *VRN2* (*VERNALIZATION 2*), and *VRN1* (*VERNALIZATION 1*), are required for the cold-mediated repression of *FLC* (Kobayashi et al., 1999; Gendall et al., 2001; Sung and Amasino, 2004). Su et al. (2018) reported that *BrVIN3.1* and *BrFLC1* are the important genetic determinants of bolting time variation in *B. rapa*. Recent reports have revealed that there are various orthologs of *FLC* and *FT* for flowering time variation in *B. rapa* and *B. oleracea* (Schiessl et al., 2017). In *B. rapa*, there are two copies of *FT* on chromosomes A02 and A07 and four copies of *FLC* on chromosomes A02, A03, and A10 (reviewed in Schiessl et al., 2017). Moreover, Shu et al. (2018) reported a major quantitative trait locus (QTL) (*Ef2.1*) for early flowering in “broccoli × cabbage” and identified *BolGRF6* as a putative candidate for early flowering in broccoli. *BrSDG8* code for encoding a histone methyltransferase is associated with bolting in *B. rapa* ssp. *pekinensis* (Fu et al., 2020). Huang et al. (2020) found that histone methyltransferase *CURLY LEAF* (*CLF*; Bra032169) controls the expression of flowering-related genes, and mutation in the Bra032169 caused early bolting in Chinese cabbage.

Gibberellic acid (GA), a plant hormone, plays an important role in the regulation of flowering time (Bernier, 1988). In *Arabidopsis*, the application of exogenous GA promotes flowering under short-day conditions (Langridge, 1957; Chandler and Dean, 1994). Mutations in genes related to GA biosynthesis and signaling can alter flowering time in *Arabidopsis* (Wilson et al., 1992; Sun and Kamiya, 1994; Jacobsen et al., 1996). In *Brassica napus*, flower initiation, flowering time, and shoot elongation are regulated by endogenous GA (Dahanayake and Galwey, 1999). Exogenous GA can stimulate flower development, while an inhibitor of GA delays or inhibits flowering in *Brassica* (Rood et al., 1989; Zanewich et al., 1990).

In flowering Chinese cabbage, aerial vegetative parts and floral buds are consumed. The early-bolting flowering Chinese cabbage double haploid (DH) line “CX14-1” is characterized by rapid flower stalk development and early flowering. In contrast, late-bolting heading Chinese cabbage exhibits a long period of vegetative growth before flower bud initiation. In *B. rapa*, long-day conditions together with vernalization promote reproductive growth over vegetative growth; consequently, plants bolt before reaching the harvesting stage, which is a serious problem in this crop (Zhang et al., 2015). Therefore, late-bolting traits are desirable than early flowering for the cultivation of spring-sown Chinese cabbage.

In this study, we performed transcriptome sequencing of flower bud samples from early-bolting flowering Chinese cabbage (“CX14-1”) (*B. rapa* ssp. *chinensis* var. *parachinensis*) and late-bolting (“Y410-1” and “SY2004”) heading Chinese cabbage (*B. rapa* var. *pekinensis*) DH lines using the Illumina platform, with a focus on genes related to plant hormone signaling.

## Materials and Methods

### Plant Materials

**FIGURE 1:**
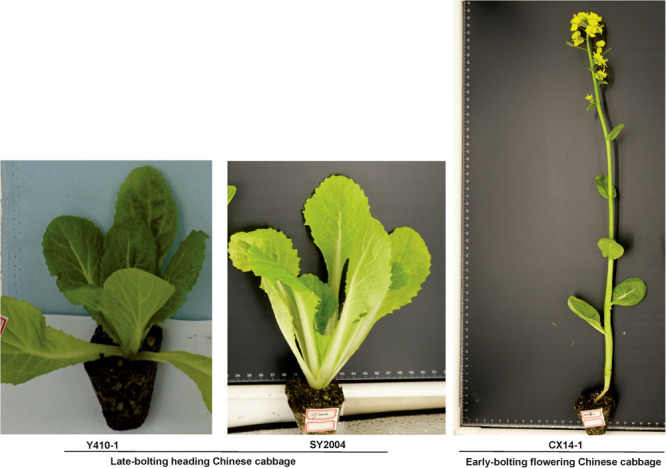
Phenotypes of early- and late-bolting Chinese cabbage double haploid lines used in the study.

The late-bolting DH heading Chinese cabbage (*B. rapa* L. ssp. *pekinensis*) lines “Y410-1” and “SY2004” and a vernalization-independent early-bolting flowering Chinese cabbage (*B. rapa* ssp. *chinensis* var. *parachinensis*) DH line “CX14-1” were used (Figure 1). The plants were grown in the experimental field at Yuanyang (113° 97’ E and 35° 5’ N), Henan Academy of Agricultural Sciences, China. The flower primordia of these lines were used for RNA sequencing. The samples were collected, immediately frozen in liquid nitrogen, and stored at -80°C.

### Total RNA Isolation, Library Construction, RNA Sequencing, and Assembly

The frozen flower bud samples were ground into a powder in liquid nitrogen. Thereafter, total RNA was isolated from 100 mg of powder using the RNeasy Mini Kit (Qiagen, Valencia, CA, United States) according to the manufacturer’s guidelines. The concentration, integrity, and purity of the RNA samples were determined using a NanoDrop spectrophotometer (NanoDrop Technologies, Wilmington, DE, United States) and Agilent 2100 Bio Analyzer (Agilent Technologies, Palo Alto, CA, United States). RNA samples with more than seven RNA integrity numbers (RINs) were used for RNA-seq library preparation with biological replications. Nine RNA-seq libraries were constructed using the NEBNext Ultra RNA Library Prep Kit for Illumina (New England Biolabs, Beverly, MA, United States) following the manufacturer’s recommendations. The clustering of the index-coded samples was performed on a cBot Cluster Generation System using the TruSeq PE Cluster Kit v4-cBot-HS (Illumina, San Diego, CA, United States) according to the manufacturer’s instructions. After cluster generation, the library preparations were sequenced on the Illumina high-throughput sequencing-by-synthesis technology platform to generate paired-end reads. The adapter sequences and low-quality reads were removed. The clean reads were then mapped to the *B. rapa* (Chiifu-401) v. 3.0 reference genome [*Brassica* database (BRAD)]^1^ using HISAT2^2^.

### Functional Annotation and Novel Gene Discovery

The functions of assembled genes and novel gene discovery were performed by BLAST searches against the NR [National Center for Biotechnology Information (NCBI) non-redundant protein sequences^3^ ], Swiss-Prot^4^, gene ontology (GO)^5^, Clusters of Orthologous Groups of proteins (COG)^6^, Pfam^7^, and Kyoto Encyclopedia of Genes and Genomes (KEGG)^8^ databases. After prediction of the amino acid sequences of the new genes, HMMER (Eddy, 1998) was used for comparisons with the Pfam database to obtain annotation information.

### Quantification of Gene Expression and Identification of Differentially Expressed Genes

Gene expression levels were estimated by fragments per kilobase of transcript per million fragments mapped (FPKM). The FPKM value for each gene was quantified according to the length of the gene and read count mapped to this gene. Differentially expressed genes (DEGs) between early-bolting and late-bolting Chinese cabbage lines were identified using the R package DEGseq (Wang et al., 2010). The resulting *p*-values were adjusted using the Benjamini and Hochberg method to control the false discovery rate. Genes with an adjusted *p*-value < 0.01 found by DEseq were identified as DEGs.

### Gene Ontology and Kyoto Encyclopedia of Genes and Genomes Pathway Enrichment Analysis

Gene ontology enrichment analysis of the DEGs was implemented by the GOseq R packages based on the Wallenius non-central hyper-geometric distribution (Young et al., 2010), which can adjust for gene length bias in DEGs. KEGG (Kanehisa et al., 2008) is a database resource for understanding high-level functions and utilities of the biological system from molecular-level information, especially large-scale molecular datasets generated by genome sequencing and other high-throughput experimental technologies (see text foot note 8). We used KOBAS (Mao et al., 2005) software to test the statistical enrichment of differential expression genes in KEGG pathways. A GO enrichment analysis of DEGs was performed using the GOseq R package based on the Wallenius non-central hyper-geometric distribution (Young et al., 2010), which can adjust for gene length bias in DEGs.

### cDNA Synthesis and qPCR Validation

A total of 1 μg of RNA was used for cDNA synthesis using SuperScript III following the manufacturer’s protocol (Invitrogen, Gaithersburg, MD, United States). Then, the FPKM values for nine randomly selected genes were validated by reverse transcription quantitative real-time PCR (RT-qPCR) using the LightCycler 480II (Roche, Mannheim, Germany). A total of 45 ng of cDNA was used as a template for RT-qPCR with gene-specific primers (Supplementary Table 1) using 2 × SyGreen Mix (qPCRBIO Lo-ROX) (PCR Biosystems, London, United Kingdom). Thermocycling conditions were 95°C for 5 min, 45 cycles of 95°C for 10 s, 60°C for 10 s, and 72°C for 15 s. At the end of the PCR cycles, the Ct values were analyzed using LightCycler 480II software (Roche). The efficiency of each gene-specific primer was determined using pooled cDNA samples. The expression of each gene was normalized using the comparative 2^–ΔΔCt^ method (Livak and Schmittgen, 2001) with *BrActin* as a reference gene.

## Results

### Transcriptome Sequencing of Early- and Late-Bolting Chinese Cabbage Floral Buds

We performed transcriptome analyses of two late-bolting heading Chinese cabbage (*B. rapa* L. ssp. *pekinensis*) DH lines (Y410-1 and SY2004) and one early-bolting flowering Chinese cabbage DH line (CX14-1) (*B. rapa* ssp. *chinensis* var. *parachinensis*) with three biological replications using Illumina sequencing technology. The raw reads generated by RNA-seq were deposited in the NCBI “Sequence Reads Archive” (SRA) under the accession number PRJNA605481. After removal of low-quality sequences, adapters, and ambiguous reads, a total of 721.49 million clean paired-end reads were obtained (Table 1). The clean reads for each sample totaled 8.81 Gb, and the Q30 base percentage was 92.71% or greater (Table 1). The clean reads were aligned with the *B. rapa* (Chiifu-401) v. 3.0 reference genome (BRAD, see text foot note 1), and the efficiency of the alignment ranged from 86.20 to 89.54%. Furthermore, variable splicing prediction, a gene structure analysis, and new gene discovery were performed based on the comparison. The clean reads were then assembled into 47,363 transcripts and 47,363 genes, of which 3,144 were predicted as novel genes (Supplementary Table 2).

**TABLE 1:** Overview of the Chinese cabbage transcriptome sequencing and assembly.

Samples	Y410-1			SY2004			CX14-1		
	T1	T2	T3	T4	T5	T6	T7	T8	T9
Clean reads	84,649,952	110,937,646	86,119,332	61,878,880	58,846,612	62,047,916	82,928,554	89,056,338	85,030,426
Clean bases	12.62	16.56	12.85	9.23	8.81	9.27	12.39	13.26	12.67
GC content (%)	47.56%	47.65%	47.75%	47.56%	47.47%	47.60%	47.39%	47.21%	47.25%
Q30 (%)	94.57%	94.75%	94.41%	92.76%	92.81%	92.71%	95.22%	94.56%	94.75%
Mapped reads	73,679,588 (87.04%)	97,117,048 (87.54%)	75,349,891 (87.49%)	54,591,125 (88.22%)	52,692,240 (89.54%)	55,186,834 (88.94%)	72,209,297 (87.07%)	76,873,854 (86.32%)	73,294,494 (86.20%)
Uniquely mapped reads	72,019,424 (85.08%)	94,886,164 (85.53%)	73,661,268 (85.53%)	53,399,380 (86.30%)	51,512,996 (87.54%)	53,981,346 (87.00%)	70,403,562 (84.90%)	74,887,781 (84.09%)	71,481,831 (84.07%)
Multiple map reads	1,660,164 (1.96%)	2,230,884 (2.01%)	1,688,623 (1.96%)	1,191,745 (1.93%)	1,179,244 (2.00%)	1,205,488 (1.94%)	1,805,735 (2.18%)	1,986,073 (2.23%)	1,812,663 (2.13%)
Reads map to “+”	36,562,440 (43.19%)	48,204,505 (43.45%)	37,387,481 (43.41%)	27,067,998 (43.74%)	26,126,686 (44.40%)	27,361,985 (44.10%)	35,865,686 (43.25%)	38,167,001 (42.86%)	36,400,366 (42.81%)
Reads map to “-”	36,694,556 (43.35%)	48,376,741 (43.61%)	37,538,457 (43.59%)	27,220,998 (43.99%)	26,275,023 (44.65%)	27,524,467 (44.36%)	35,975,214 (43.38%)	38,287,253 (42.99%)	36,504,731 (42.93%)

*Q30 is the percentage of bases within a Phred value > 30.*

### Identification of Differentially Expressed Genes

**FIGURE 2:**
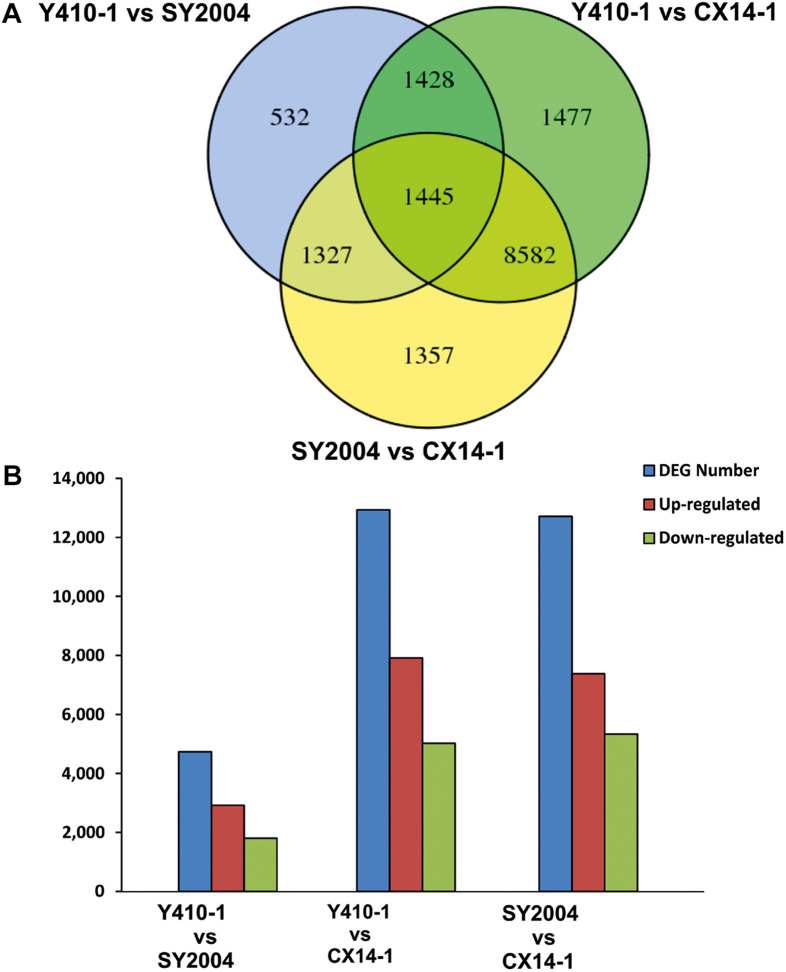
Differentially expressed genes (DEGs) between early- and late-bolting Chinese cabbage double haploid lines. **(A)** Venn diagram DEGs identified through pairwise comparisons. **(B)** Number of up- and downregulated genes in each comparison. Venn diagram was generated using the freely available VENNY 2.1 online tool (http://bioinfogp.cnb.csic.es/tools/venny/).

**FIGURE 3:**
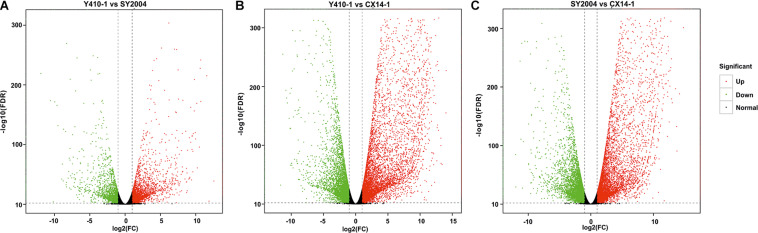
Volcano plots of differentially expressed genes (DEGs) for each comparison in flower bud of early- and late-bolting Chinese cabbage double haploid lines. The *x* and *y* axes indicate logarithm fold change [log_2_(FC)] of the difference in the expression of a gene between two samples and the negative logarithm of the statistical significance [log_2_(FDR)] of the change in gene expression, respectively. The red and green dots represent significantly up- and downregulated genes, respectively; while black dots represent non-differentiated genes. FC, fold change; FDR, false discovery rate.

**FIGURE 4:**
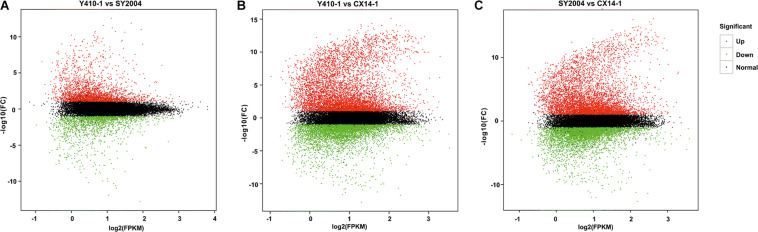
MA plot of differential expression for two samples. Each point represents the differentially expressed genes (DEGs). The *x* axis indicates log_2_(FPKM), and the *y* axis indicates log_2_(FC). The red and green dots represent significantly up- and downregulated genes, respectively; while black dots represent non-differentiated genes. FPKM, fragments per kilobase of transcript per million fragments mapped; FC, fold change.

**FIGURE 5:**
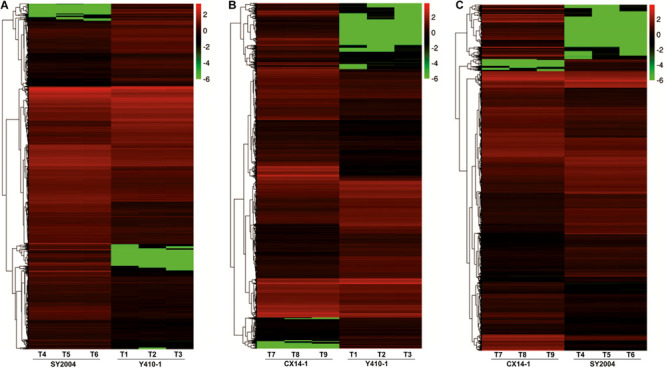
Heatmap representation of differentially expressed genes (DEGs) in the floral bud of early- and late-bolting Chinese cabbage double haploid lines. The expression patterns are based on the log_2_ of FPKM (fragments per kilobase of transcript per million fragments mapped for each gene) used for hierarchical clustering for each sample. The different columns represent different samples, with different rows representing different genes. Biological replicates: T1, T2, and T3 (Y410-1); T4, T5, and T6 (SY2004); and T7, T8, and T9 (CX14-1).

A total of 30,375 DEGs (Supplementary Tables 3–5) were identified in pairwise comparisons (Y410-1 vs. SY2004, Y410-1 vs. CX14-1, and SY2004 vs. CX14-1) (Figure 2A). The most DEGs were found in Y410-1 vs. CX14-1 (12,932), with 7,913 upregulated and 5,019 downregulated genes (Figure 2). The fewest DEGs were found in Y410-1 vs. SY2004 (4732), with 2,925 and 1,807 up- and downregulated genes, respectively (Figure 2B). Overall, 1,445 DEGs were common to all comparisons. Volcano plots (Figure 3) and MA plots (Figure 4) were generated to summarize significant DEGs. The upregulated and downregulated genes in comparisons between each pair of early-bolting Chinese cabbage lines were determined by hierarchical clustering based on FPKM values (Figure 5).

### Functional Annotation and Classification

**FIGURE 6:**
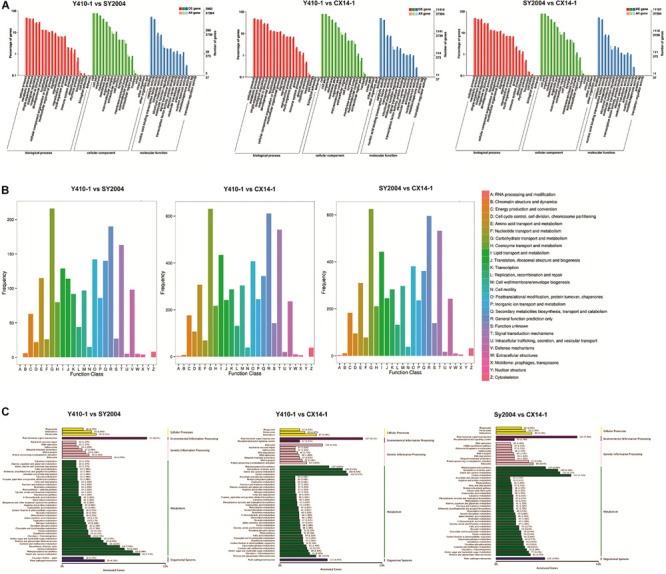
Functional classification of differentially expressed genes (DEGs) among early- and late-bolting Chinese cabbage double haploid lines. **(A)** Gene ontology (GO), **(B)** Clusters of Orthologous Groups of proteins (COG), and **(C)** Kyoto Encyclopedia of Genes and Genomes (KEGG) classification of DEGs.

**FIGURE 7:**
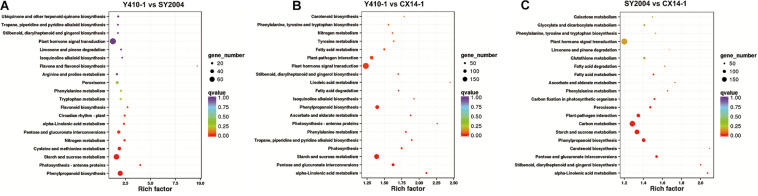
Kyoto Encyclopedia of Genes and Genomes (KEGG) pathway enrichment scatter plot of differentially expressed genes. Rich factor is the ratio of the differentially expressed gene number to the total gene number in a certain pathway. The dot size and color indicate the number of genes and the range of the false discovery rate (FDR) value, respectively.

The putative functions of assembled genes were annotated by searches against public databases, including GO, COG, KEGG Orthology (KOG), KEGG, eggNOG, PFAM, NR, and SWISS-PROT. Among them, 47,214, 40,497, 35, 083, 32,944, and 24,390 genes were annotated to the NR, eggNOG, PFAM, SWISS-PROT, and KOG databases, respectively (Table 2). In addition, 2,564 of 3,144 novel genes were functionally annotated (Supplementary Table 2). Furthermore, we performed GO, COG, and KEGG pathway analyses to illustrate the biological functions of the Chinese cabbage floral bud transcriptomes. A GO term enrichment analysis was performed to identify terms in three general categories, biological process (BP), molecular function (MF), and cellular component (CC) (Berardini et al., 2004) (Figure 6A). A total of 37,394 genes were assigned to 54 main functional groups. The cellular process (GO:0009987), metabolic process (GO:0008152), and single-organism process (GO:0044699) were the most important in the BP category. Cell (GO:0005623), cell part (GO:0044464), and organelle (GO:0043226) were highly enriched in CC. Binding (GO:0005488) and catalytic activity (GO:0003824) were the most important GO terms in MF (Supplementary Table 6–8 and Figure 6A). These results indicated that early and late bolting might be associated with DEGs in these functional subgroups. According to a COG functional annotation analysis, 14,411 genes were classified into 25 COG categories (Figure 6B). The predominant COG categories represented in the Chinese cabbage floral bud transcriptomes were G (carbohydrate transport and metabolism), R (general function prediction only), and T (signal transduction mechanisms) (Figure 7B). Several genes in these categories were differentially expressed, which might contribute to flowering time differences between the early- and late-bolting Chinese cabbage DH lines.

**TABLE 2:** Summary of functional annotation and classification of assembled genes.

Database annotated	No. of genes	Length (300 ≤ 1,000)	Length (≥1,000)
COG	14,411	4,341	9,839
GO	37,394	15,993	19,336
KEGG	15,408	6,204	8,422
KOG	24,390	9,639	13,624
Pfam	35,083	14,009	20,085
Swiss-Prot	32,944	13,405	18,021
eggnog	40,497	17,383	20,916
NR	47,214	21,202	22,487
All	47,363	21,268	22,505

*COG, Clusters of Orthologous Groups of proteins; GO, Gene Ontology; KEGG, Kyoto Encyclopedia of Genes and Genomes; KOG, KEGG Orthology.*

### Kyoto Encyclopedia of Genes and Genomes Pathway Enrichment Analysis of the Differentially Expressed Genes

Different gene products interact with each other to exert biological functions, and pathway annotation analyses are therefore useful to predict the functions of gene. KEGG is a resource for the systematic analysis of gene function and genomic information databases, providing information about genes and expression patterns from a whole network perspective (Kanehisa et al., 2004). We therefore analyzed the metabolic pathways of the DEGs between early- and late-bolting Chinese cabbage lines using the KEGG database. Total 2,430 and 2,417 DEGs were assigned to 124, 128, and 127 pathways for Y410-1 vs. SY2004, Y410-1 vs. CX14-1, and SY2004 vs. CX14-1, respectively (Supplementary Table 9). Moreover, these pathways involved 15,408 genes, which were different from the DEGs assigned to pathways, indicating that some genes contribute to more than one KEGG pathway (Table 2 and Supplementary Table 9). For example, the novel gene predicted in this study “Brassica_rapa_newGene_2848” is involved in homologous recombination, mismatch repair, nucleotide excision repair, and DNA replication. However, six pathways, including plant–pathogen interaction, starch and sucrose metabolism, carbon metabolism, biosynthesis of amino acids, phenylpropanoid biosynthesis, and plant hormone signal transduction, contained over 100 DEGs between early- and late-bolting lines (SY410-1 vs. CX14-1 and SY2004 vs. CX14-1) (Figure 6C). Further, a KEGG pathway enrichment analysis of DEGs between early-and late-bolting lines revealed that plant hormone and signal transduction (197 genes) (Ko04075), starch and sucrose metabolism (158 genes) (Ko00500), and phenylalanine biosynthesis (117 genes) (Ko00940) are highly enriched for Y410-1 vs. CX14-1, while carbon metabolism (177 genes) (Ko01200), plant hormone and signal transduction (191 genes) (Ko04075), starch and sucrose metabolism (151 genes) (Ko00500), and phenylalanine biosynthesis (117 genes) (Ko00940) are highly enriched for SY2004 vs. CX14-1 (Figure 7 and Supplementary Table 8). Plant hormone and signal transduction as well as starch and sucrose metabolism were common to both comparisons. Among genes related to plant hormone and signal transduction, 98 and 99 genes were up- and downregulated in Y410-1 vs. CX14-1 (Supplementary Table 10), and 89 and 102 genes were up- and downregulated in SY2004 vs. CX14-1 (Supplementary Table 10).

### Expression Patterns of Differentially Expressed Genes Related to Plant Hormone and Signal Transduction

**FIGURE 8:**
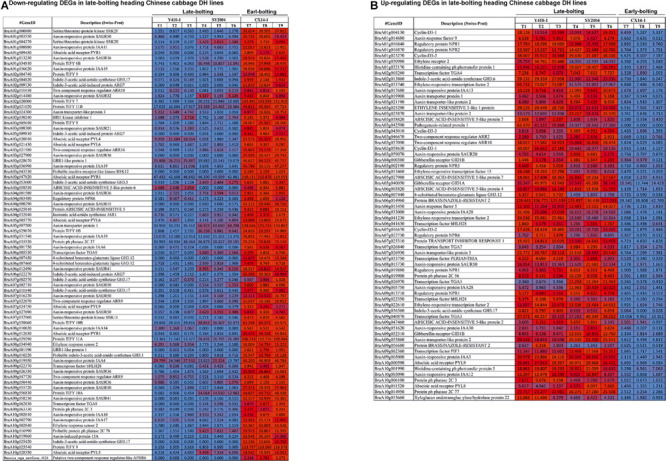
Heatmap representation of the expression pattern [fragments per kilobase of transcript per million fragments mapped (FPKM) values] of differentially expressed genes (DEGs) related to plant hormone and signal transduction. **(A)** Downregulated and **(B)** upregulated DEGs in both late-bolting heading Chinese cabbage double haploid (DH) lines.

The expression levels of most of the DEGs related to ABA receptors were downregulated, except *PYL8* and *PYL9*, while most of the DEGs encoding ABA-insensitive five-like protein were upregulated in late-bolting Y410-1 compared with early-bolting flowering Chinese cabbage DH lines (Figure 8 and Supplementary Table 9). Similarly, the expression levels of most of the DEGs related to ABA receptors were downregulated, except for *PYL8* and *PYL9*. In contrast, most of the DEGs related to ABA-insensitive 5-like proteins were upregulated in late-bolting compared with early-bolting flowering Chinese cabbage DH lines (Figure 8 and Supplementary Table 10).

The expression levels of DEGs encoding gibberellin receptors GID1A and GID1B were upregulated in both late-bolting (Y410-1 and SY2004) heading Chinese cabbage compared with the early-bolting flowering Chinese cabbage DH line (Figure 8 and Supplementary Table 10).

The expression levels of JA signaling genes, like *jasmonate-amino synthetase* (*JAR1*, a GH3 family of protein), were downregulated in both late-bolting heading Chinese cabbage DH lines (Y410-1 and SY2004) compared with the early-bolting flowering Chinese cabbage DH line (Figure 8 and Supplementary Table 10). Moreover, the expression levels of DEGs encoding TIFY proteins were upregulated in the early-bolting flowering Chinese cabbage DH line (CX14-1) compared with both late-bolting heading Chinese cabbage lines (Figure 8 and Supplementary Table 10).

The expression levels of DEGs encoding brassinazole-resistant 2 proteins (*BraA09g056680*, *BraA06g014960*, and *BraA08g028240*) and brassinosteroid-insensitive 1-associated receptor kinase 1 (*BraA08g016610*) were upregulated, whereas BRI1 kinase inhibitor 1 (*BraA02g030240*) was downregulated in both late-bolting heading Chinese cabbage lines compared with early-bolting flowering Chinese cabbage DH lines (Figure 8 and Supplementary Table 9). Furthermore, two DEGs related to DELLA protein RGA1 (*BraA06g040430*) and RGA2 (*BraA09g023210*) were upregulated in both late-bolting heading Chinese cabbage lines.

In case of ethylene signaling genes, DEGs encoding ethylene response sensors, such as *ethylene response sensor 2* and *ethylene-insensitive 3-like 3*, were downregulated, while *ethylene-responsive transcription factor 2* (*BraA06g041230*, *BraA02g033740*, and *BraA09g022610*), *ethylene-responsive transcription factor 15* (*BraA05g013460* and *BraA04g022530*), and ethylene receptors, such as *ethylene-insensitive 3-like 1* (*BraA03g025290*), were upregulated in both late-bolting heading Chinese cabbage DH lines compared with the early-bolting flowering Chinese cabbage DH line (Figure 8 and Supplementary Table 10).

The unigenes related to auxin signal transduction, including auxin-responsive proteins, auxin transporter-like proteins, auxin responsive factor (ARF), and auxin-induced proteins, were differentially expressed between late-bolting heading Chinese cabbage and early-bolting flowering Chinese cabbage DH lines. The expression levels of 34 of these DEGs were upregulated in late-bolting heading Chinese cabbage Y410-1 compared with early-bolting flowering Chinese cabbage DH lines. Likewise, 27 and 33 DEGs were down- and upregulated, respectively, in another late-bolting DH line, SY2004 (Figure 8 and Supplementary Table 10). However, among these DEGs, 27 upregulated and 15 downregulated were common to both late-bolting lines (Supplementary Table 10).

### Expression Patterns of Differentially Expressed Genes Related to Starch and Sucrose Metabolism

**FIGURE 9:**
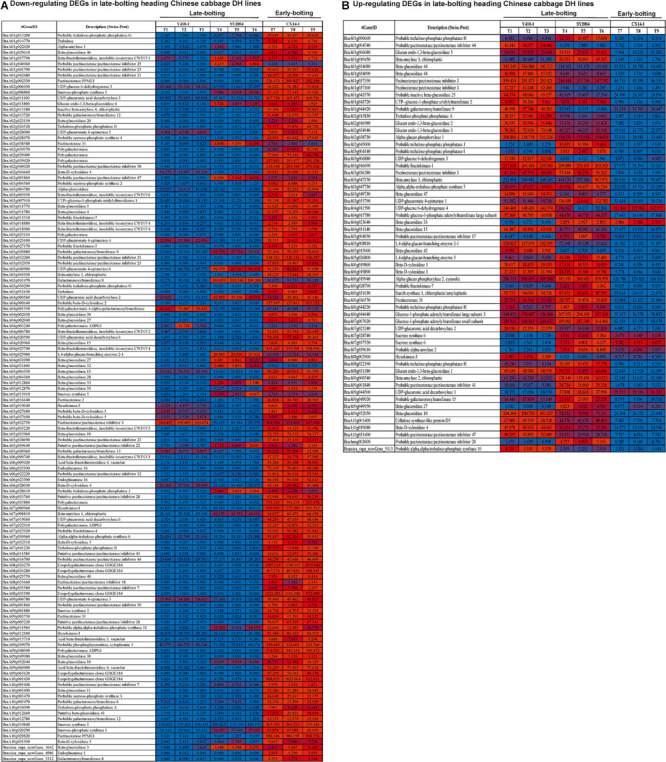
Heatmap representation of the expression pattern [fragments per kilobase of transcript per million fragments mapped (FPKM) values] of differentially expressed genes (DEGs) related to starch and sucrose metabolism. **(A)** Downregulated and **(B)** upregulated DEGs in both late-bolting heading Chinese cabbage double haploid (DH) lines.

Among the DEGs related to starch and sucrose metabolism, trehalose-phosphate phosphatase (A, B, G, and J), trehalase, alpha-amylase, beta-glucosidase, beta-fructofuranosidase, pectinesterase/pectinesterase inhibitor, sucrose-phosphate synthase, UDP-glucuronic acid decarboxylase, inactive beta-amylase, galacturonosyltransferase, UDP-glucuronate 4-epimerase, polygalacturonase, beta-D-xylosidase (2, 3, 5), alpha-glucosidase, UTP-glucose-1-phosphate uridylyltransferase 1, fructokinase (4, 5, 7), galacturonosyltransferase, polygalacturonate 4-alpha-galacturonosyltransferase, 1,4-alpha-glucan-branching enzyme, hexokinase, acid beta-fructofuranosidase, endoglucanase, alpha-trehalose-phosphate synthase, and exopolygalacturonase were downregulated in both late-bolting heading Chinese cabbage DH lines (Y410-1 and SY2004) compared with the early-bolting flowering Chinese cabbage (Figure 9). On the other hand, trehalose-phosphate phosphatase (I, H), glucan endo-1,3-beta-glucosidase, UTP-glucose-1-phosphate uridylyltransferase 2, alpha-glucan phosphorylase (1, 2), fructokinase-1, glucose-1-phosphate adenylyltransferase large subunit, beta-D-xylosidase (1, 4), starch synthase 1, and cellulose synthase-like protein D5 were upregulated in both late-bolting heading Chinese cabbage lines (Figure 9).

### Validation of RNA-seq Data by RT-qPCR

**FIGURE 10:**
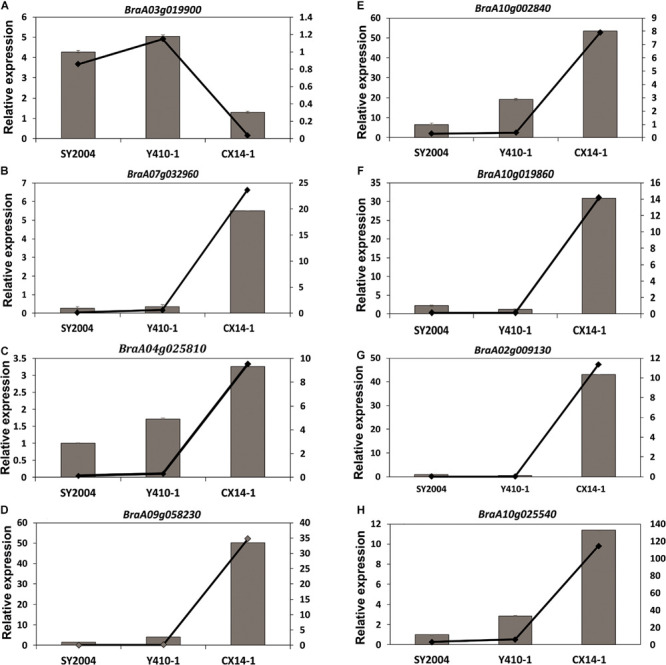
Relative expression of eight unigenes in the floral bud of early- and late-bolting Chinese cabbage double haploid lines. Error bar represents ± SE of the means of triplicates. The superimposed line graph represents the RNA-seq expression profiles [fragments per kilobase of transcript per million fragments mapped (FPKM)].

We further tested the reliability of FPKM expression patterns (determined by RNA-seq) by RT-qPCR. Eight DEGs were randomly selected, and their relative expression levels were quantified using the same RNA samples extracted from early- and late-bolting Chinese cabbage lines for RNA sequencing. The results confirmed that the expression patterns of the analyzed unigenes were consistent with the FPKM expression pattern obtained by RNA-seq (Figure 10).

## Discussion

Late bolting is an important economic trait in spring-sown lines, and late-bolting varieties have been developed and characterized for off-season production to meet the annual demand for Chinese cabbage (Yang et al., 2007; Su et al., 2018). Nevertheless, little is known about the genes and pathways associated with flowering time in flowering Chinese cabbage (*B. rapa* ssp. *chinensis* var. *parachinensis*) and late-bolting heading Chinese cabbage (*B. rapa* var. *pekinensis*). RNA sequencing technology has been effectively utilized for transcriptome analyses in a wide range of texa (Wang et al., 2009; Lu et al., 2010). This high-throughput sequencing technology can be used to determine global gene expression differences between populations or species with phenotypic differences and responses to environmental stress (Mortazavi et al., 2008; Wang et al., 2009; Miao and Luo, 2013). In the present study, we performed a comparative transcriptome analysis of early-bolting Chinese cabbage DH lines by RNA-seq. We obtained 721.49 million clean paired-end reads assembled into 47,363 unigenes, including 3,144 genes predicted as novel (Table 1 and Supplementary Table 2). We detected 12,932 (Y410-1 vs. CX14-1) and 12,711 (SY2004 vs. CX14-1) DEGs between two sets of early- and late-bolting Chinese cabbage (Figure 2A and Supplementary Tables 4, 5).

A functional analysis of assembled unigenes revealed that the predominant COG categories were carbohydrate transport and metabolism, general function prediction only, and signal transduction mechanisms (Figure 6B). Several unigenes in these categories were differentially expressed and might explain the difference in flowering times in early-bolting Chinese cabbage DH lines. Moreover, we identified 3,144 novel unigenes, of which 2,564 were functionally annotated (Supplementary Table 2).

A KEGG pathway enrichment analysis of DEGs between early- and late-bolting DH lines indicated that plant hormone and signal transduction (Ko04075) and starch and sucrose metabolism (Ko00500) were the most enriched pathways in both comparisons (Supplementary Table 9). These results suggest that unigenes related to plant hormones, signal transduction, and sucrose metabolism are involved in the regulation of flowering time in these Chinese cabbage DH lines.

The “*no hydrotropic response*” (*nhr1*) *Arabidopsis* mutants show high levels of ABA, resulting in late flowering (Quiroz-Figueroa et al., 2010), whereas ABA-deficient (*aba2* and *aba3*) or ABA-insensitive (*abai4*) mutants show early flowering than wild types (Martínez-Zapater et al., 1994; Matsoukas, 2014). Moreover, Zhu et al. (2013) demonstrated that *ABA-insensitive 5* (*ABI5*, encoding a bZIP transcription factor) delayed flowering time in *Arabidopsis* under long-day conditions. Our results also indicated that the expression levels of most genes encoding ABI5-like proteins (ABA-insensitive 5-like protein 2, ABA-insensitive 5-like protein 4, ABA-insensitive 5-like protein 5, ABA-insensitive 5-like protein 6, and ABA-insensitive 5-like protein 7) were higher in late-bolting Chinese cabbage DH lines than in early-bolting flowering Chinese cabbage (Figure 8 and Supplementary Table 10).

Previous studies of mutants related to GA biosynthesis or signal transduction have revealed that GA can alter the flowering time (Wilson et al., 1992; Peng and Harberd, 1993; Sun and Kamiya, 1994; Jacobsen et al., 1996; Peng et al., 1997; Andres et al., 2014). Moreover, the DELLA domain protein RGA (repressor of ga1-3), GAI (GA insensitive), and RGA-like1 (RGL1 and RGL2) act as negative regulators of the GA signaling pathway (Yu et al., 2004; Silverstone et al., 2007). Our results revealed that two DEGs encoding DELLA proteins RGA1 (BraA06g040430) and RGA2 (BraA09g023210) are upregulated in late-bolting heading Chinese cabbage “Y410-1” (Figure 8 and Supplementary Table 10) and downregulated at the flowering stage compared with the vegetative stages in early-bolting flowering Chinese cabbage. These results suggest that the downregulation of negative regulators of the GA signaling pathway might increase GA levels, and the lack of function of these genes may trigger early bolting. Similar results have been reported by Huang et al. (2017), who reported that *RGA1* and *RGA2* are downregulated in flowering Chinese cabbage.

Several previous studies have indicated that the phytohormone jasmonic acid (JA) also regulates flowering time in *Arabidopsis* (Zhai et al., 2015). The F-box protein COI1 (coronatine insensitive 1) degrades JAZ (contains TIFY and Jas domains) repressors (Hoo and Howe, 2009). Transcript levels of *NaJAZd* and *NaJAZh* are upregulated in the early floral stages of *NaJAZi-*silenced plants due to the high JA content in the flowers (Li et al., 2017). Our results also showed that DEGs encoding TIFY proteins are more highly expressed in the early-bolting flowering Chinese cabbage DH line (CX14-1) than in both late-bolting heading Chinese cabbage lines (Figure 8 and Supplementary Table 10). These results suggest that the elevated expression of these genes in early flowering Chinese cabbage might be related to a high JA content.

Another steroidal phytohormone, brassinosteroid (BR), promotes flower induction in plants (Matsoukas, 2014). The BR signaling genes *BZR1* (*brassinazole-resistant1*) and *BES1* suppress the expression of important genes related to BR biosynthesis (*CPD*, *constitutive photomorphogenesis, and dwarfism*; *DWF4*, and *DWARF4*) by binding to their promoter regions in *Arabidopsis* (Wei et al., 2017). Moreover, BR-deficient/BR-insensitive mutants show delayed flowering time (Domagalska et al., 2007; Zhu et al., 2013). We also found that DEGs related to *BZR1*, *BZR2*, and *BRI1* (*brassinosteroid insensitive 1*) were more highly expressed in both late-bolting heading Chinese cabbage DH lines than in early-bolting flowering Chinese cabbage (Figure 8 and Supplementary Table 10). These results suggest that the upregulation of BR signaling genes might affect BR biosynthesis and cause late bolting in late-bolting heading Chinese cabbage DH lines.

Starch and sucrose play important roles in flowering (Turnbull, 2011; Cho et al., 2018). Previous reports revealed that trehalose-6-phosphate acts as a signal molecule for flowering initiation in different plant species, including *Arabidopsis thaliana* (Sheen, 2014), grape (Caspari et al., 1998), and citrus (Shalom et al., 2014). Besides, Micallef et al. (1995) found that an increased level of endogenous sucrose promotes flowering in tomato. Wahl et al. (2013) reported that *TREHALOSE-6-PHOSPHATE SYNTHASE 1* (*TPS1*) is required for the regulation of flowering time in *A. thaliana*. In the present study, *alpha*,*alpha-trehalose-phosphate synthase 5* (BraA03g047730) was upregulated in both late-bolting heading Chinese cabbage lines compared with early-bolting flowering Chinese cabbage lines (Figure 9).

Razi et al. (2008) demonstrated that *BoFLC* alleles segregate independently from flowering time alleles in *Brassica oleracea*. However, Yuan et al. (2009) reported that variation in *BrFLC1* is linked to flowering time in *B. rapa*. Moreover, Xie et al. (2015) performed a QTL analysis and showed that *Br2* is the key determinant of *BrFLC2* and a candidate flowering time locus in *B. rapa*. A transposon insertion in the coding sequence of *BrFT2* located on a QTL on chromosome A07 (region Br5) causes late flowering (Zhang et al., 2015). Therefore, we further analyzed the flowering time-related genes, especially *FLC* and *FT*, based on FPKM expression values. Unigenes, such as *FLK* (*BraA03g031700*) and *FLD* (*BraA03g034300*), showed higher expression levels in both late-bolting lines than in early-bolting lines, while *FLT* (*BraA02g016700*) showed the opposite pattern (Supplementary Table 11). With regard to *FT* genes, the expression levels of three unigenes encoding proteins related to flowering time control, such as FCA (BraA01g020520), FY (BraA02g004930), and FPA (BraA09g036880), were higher in both late-bolting lines than in early-bolting lines (Supplementary Table 11). Nonetheless, none of these unigenes were differentially expressed between early- and late-bolting lines.

In late-bolting Chinese cabbage, 98 and 89 unigenes related to plant hormone and signal transduction were upregulated in Y410-1 vs. CX14-1 and SY2004 vs. CX14-1, respectively (Figure 8 and Supplementary Table 9). Among these, 19 and 12 unigenes showed log_2_ fold change values of > 5.0 in Y410-1 vs. CX14-1 and SY2004 vs. CX14-1, respectively; while six unigenes, including *BraA02g009130* (indole-3-acetic acid-induced protein ARG7), *BraA09g058230* (Auxin-responsive protein SAUR41), *BraA07g032960* (serine/threonine-protein kinase BSK11), *BraA10g019860* (auxin-induced protein 15A), *BraA08g012630* (abscisic acid receptor PYR1), and *BraA01g009450* (abscisic acid receptor PYR1), were common in both late-bolting heading Chinese cabbage lines (Y410-1 and SY2004). These unigenes are candidates for earl-bolting and late-bolting traits in these DH Chinese cabbage lines and could be useful for the development of molecular markers for the detection of early- and late-bolting cultivars.

## Data Availability Statement

Data is available at NCBI SRA accession: PRJNA605481.

## Author Contributions

XZ and YY conceived and designed the experiments. YZ, SY, and ZW performed the experiments. HS, LL, LN, and MH-U-R prepared the figures and tables. XW and MR drafted the work or revised it critically for important content. All authors contributed to the article and approved the submitted version.

## Conflict of Interest

The authors declare that the research was conducted in the absence of any commercial or financial relationships that could be construed as a potential conflict of interest.

## Footnotes

1 http://brassicadb.org/brad/

2 http://ccb.jhu.edu/software/hisat2/index.shtml

3 ftp://ftp.ncbi.nih.gov/blast/db/

4 http://www.uniprot.org/

5 http://www.geneontology.org/

6 http://www.ncbi.nlm.nih.gov/COG/

7 http://pfam.xfam.org/

8 http://www.genome.jp/kegg/
